# Optical coherence tomography angiography of the macula and optic nerve head: microvascular density and test-retest repeatability in normal subjects

**DOI:** 10.1186/s12886-018-0976-y

**Published:** 2018-12-10

**Authors:** Ching Wei Lim, Jun Cheng, Elton Lik Tong Tay, Hwei Yee Teo, Elizabeth Poh Ying Wong, Vernon Khet Yau Yong, Boon Ang Lim, Owen Kim Hee, Hon Tym Wong, Leonard Wei Leon Yip

**Affiliations:** 1 Department of Ophthalmology, Tan Tock Seng Hospital, 11 Jalan Tan Tock Seng, Singapore, 308433 Singapore; 2 Department of Ophthalmology, Sarawak General Hospital, Jalan Hospital, 93586 Kuching, Sarawak Malaysia; 3 Ocular Imaging Department, Institute for Infocomm Research, Agency for Science, Technology and Research, Singapore, Singapore

**Keywords:** Optical coherence tomography angiography, Test-retest repeatability, Intra-observer variation, Healthy volunteers

## Abstract

**Background:**

Despite the potential usefulness of optical coherence tomography angiography in retinal and optic disc conditions, the reliability of the imaging modality remains unclear. This study set out to measure the microvascular density of macula and optic disc by mean of optical coherence tomography angiography and report the repeatability of the vessel density measurements.

**Methods:**

Cross sectional observational cohort study. Subjects with normal eyes were recruited. Two sets of optical coherence tomography angiography images of macula and optic nerve head were acquired during one visit. Novel in-house developed software was used to count the pixels in each images and to compute the microvessel density of the macula and optic disc. Data were analysed to determine the measurement repeatability.

**Results:**

A total of 176 eyes from 88 consecutive normal subjects were recruited. For macular images, the mean vessel density at superficial retina, deep retina, outer retina and choriocapillaries segment was OD 0.113 and OS 0.111, OD 0.239 and OS 0.230, OD 0.179 and OS 0.164, OD 0.237 and OS 0.215 respectively. For optic disc images, mean vessel density at vitreoretinal interface, radial peripapillary capillary, superficial nerve head and disc segment at the level of choroid were OD 0.084 and OS 0.085, OD 0.140 and OS 0.138, OD 0.216 and OS 0.209, OD 0.227 and OS 0.236 respectively. The measurement repeatability tests showed that the coefficient of variation of macular scans, for right and left eyes, ranged from 6.4 to 31.1% and 5.3 to 59.4%. Likewise, the coefficient of variation of optic disc scans, for right and left eyes, ranged from 14.3 to 77.4% and 13.5 to 75.3%.

**Conclusions:**

Optical coherence tomography angiography is a useful modality to visualise the microvasculature plexus of macula and optic nerve head. The vessel density measurement of macular scan by mean of optical coherence tomography angiography demonstrated good repeatability. The optic disc scan, on the other hand, showed a higher coefficient of variation indicating a lower measurement repeatability than macular scan. Interpretation of optical coherence tomography angiography should take into account test-retest repeatability of the imaging system.

**Trial registration:**

National Healthcare Group Domain Specific Review Board (NHG DSRB) Singapore. DSRB Reference: 2015/00301.

**Electronic supplementary material:**

The online version of this article (10.1186/s12886-018-0976-y) contains supplementary material, which is available to authorized users.

## Background

The posterior segment of the eye contains a highly complex vascular system. Pathological changes of the microvasculature in the posterior segment are associated with various eye diseases which potentially lead to blindness. In recent decades, posterior segment microvasculature imaging has become an essential tool in the diagnosis and management of many posterior segment conditions. Traditionally, the microvascular circulation in the posterior segment is visualized by means of Fundal Fluorescein Angiography (FA) and Indocyanine Green (ICG) angiography imaging systems. These imaging techniques provide important information about the posterior segment blood circulation and play a central role in the management of retinal and choroidal conditions. However, the necessity of using intravenous fluorescein or indocyanine green dye make them invasive investigations with risk of mortality and morbidity.

Optical coherence tomography angiography (OCTA) has emerged as a new innovation in ocular vasculature imaging technology. In contrast to FA and ICG, OCTA is non-contact, non-invasive, easy to perform and capable of providing a clear three dimensional image of the ocular microvascular circulation. In addition, the En Face feature of newer OCTA allows the clinician to view the microvascular plexus that are located at varying depths of posterior segment structures. A considerable amount of recent literature suggests the useful application of OCTA in retinal and choroidal pathological conditions, including diabetic retinopathy, retinal vascular disease, age-related macular degeneration, central serous chorioretinopathy, and macular microangiopathy such as sickle cells diseases [1–6]. In additional to that, several studies have also utilized OCTA to detect perfusion changes in optic nerve head in multiple sclerosis and glaucoma [7–12]. In fact, it has been suggested that OCTA is potentially an alternative to FA in posterior segment imaging [3, 13, 14].

In clinical practice, repeated posterior segment imaging is often required in diseased eyes in order to detect progression or to monitor treatment response. An imaging modality with good repeatability and reproducibility is therefore a prerequisite in deciding if the modality can be relied on in disease management. Despite the new knowledge about potential OCTA applications, there is little published data on the consistency and reliability of OCTA. Jia et al. reported that OCTA showed a low intra-visit and inter-visit flow index variability [15]. However, the study was limited to optic disc perfusion measurement and the reliability analysis was based upon data from a small number of subjects. It is unclear if retina OCTA has similar test variability and whether OCTA shows a consistent accuracy and repeatability across depths of posterior segment structures. The purpose of our study is to assess the repeatability of OCTA imaging in ocular microcirculation of a normal population and to determine the OCTA variability across varying depths of retina, choroid and optic disc.

## Methods

This cross sectional prospective observational cohort study was conducted by the Department of Ophthalmology, Tan Tock Seng Hospital (TTSH) and Institute for Infocomm Research(I2R), Agency for Science, Technology and Research, Singapore. It was approved by The Institutional Review Board of the National Healthcare Group. Written informed consent was obtained prior to enrollment according to the tenets of the Declaration of Helsinki.

Subjects were enrolled from the ophthalmology clinic of TTSH, between April 2015 to January 2016. All eligible subjects underwent a series of standard ophthalmic assessment and investigation, including visual acuity measurement, slit lamp examination, fundus examination, intraocular pressure measurement by Goldmann Applanation Tonometry and visual field examination with Humphrey visual field Analyzer SITA-Fast 24–2 (Carl Zeiss Meditec, Dublin, CA). The criteria for selecting the subjects were BCVA of 6/12 or better, normal optic disc and retina, normal visual field and intraocular pressure lower than 22 mmHg. We excluded subjects with ocular pathology (myopic degeneration, age related macular degeneration, diabetic maculopathy, optic neuropathy, glaucoma, previous laser therapy or surgery), subjects with systemic disease that can produce visual field defect, pregnant women or subjects younger than 21 years of age.

### OCTA imaging

In this study, we used the AngioVue Enhanced Microvascular Imaging System (Optovue, Fremont, CA, USA. Software version 2016.2.0.35) in OCTA image acquisition. The system is a dual-modality OCT system, which integrates spectral domain OCT and split-spectrum amplitude-decorrelation angiography (SSADA) algorithm to generate motion-contrast blood flow images. This allows 3-dimentional visualization of posterior segment microvasculature. The principle of the SSADA algorithm has been well described elsewhere [16].

We follow the manufacturer recommended scanning technique to capture images. In brief, subject was positioned in front of the AngioVue system with good eye alignment. The system internal fixation target was used to maintain steady gaze. The machine auto focus technology was used to allow accurate focus on the posterior segment structure of interest. Subjects were advised to maintain stable head position and gaze during scanning, but allowed to rest, blink or repositioning in between scans. The optic nerve head scan was a 3 x3mm cube centered on the optic nerve head. The macular scan was a 3 × 3 mm cube centred on the fovea. After each scanning process, two same observers reviewed and filtered the image captured immediately. Images with significant motion artifacts, poor image clarity or poor signal strength index (< 40) were discarded. To determine the presence of significant motion artifacts, observers put a flag on the particular segment image with obvious large motion based on visual examination of the OCTA images. High motion artifacts was defined as having more than two white lines (straight line from the left to the right of the image) in the image. In the circumstance when a segment image were discarded, other segment images from the same scan may be included for further analysis if they were of good quality.

In a subset of patients, the scanning process was repeated in order to obtain a total of 2 OCTA images of optic nerve head and macular of good quality from both eyes of each subject. The first scan of all eyes was used for demographics, clinical characteristics and vessels density analysis.

The AngioVue System automatically segmented the optic disc and macula into specific layers as below. This produced detailed en face images of the layer with clear visualization of the microvascular system of the specific layer.

1. Macula:
  A. Superficial retinal capillary plexus
  B. Deep retinal capillary plexus
  C. Outer retinal capillary plexus
  D. Choriocapillaries
2. Optic disc:
  A. Vitreoretinal interface
  B. Superficial optic nerve head
  C. Radial peripapillary capillaries (RPC) at the level of the retinal nerve fiber layer
  D. Optic disc at the level of the choroid

**Fig. 1:**
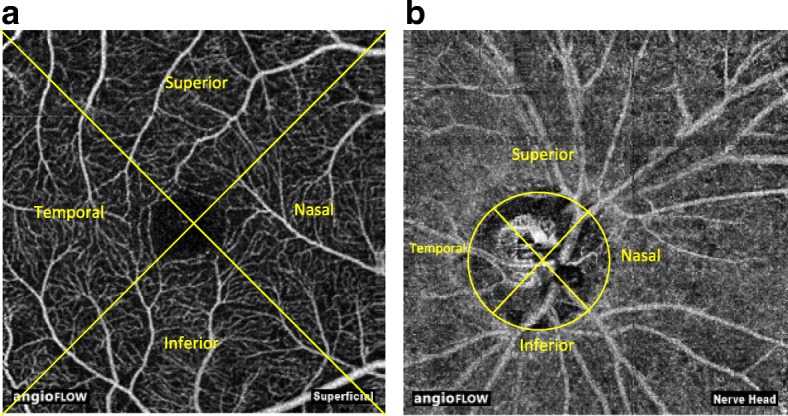
**a** Quadrant division of macular scan. **b** Quadrant division of optic disc scan

In optic disc segment images, we manually drew the minimum bounding box of the optic disc, from which an ellipse is approximated as the border of optic disc. Subsequently for both macular and optic disc scan, we subdivided each segmented layers into 4 equal quadrants, namely superior, inferior, temporal and nasal quadrant. For example, as shown in the figure blow, the four quadrants are determined based on a diagonal and anti-diagonal line (Fig. 1a and b). As the result of segmentation and further division, our study produced 4 segmented layers and 16 quadrants for macula and optic disc respectively in each eyes.

### Vessel pixel density calculation

Novel in-house developed software involving vessel detection and pixels counting was used to determine the blood vessels density of each Enface segments and quadrants of optic disc and macular for images 1 and 2. The results were presented as percentage of pixel density of the vessel against the background.

To compute vessel density, we first apply bandpass filter to suppress the horizontal noise lines caused by eye movement using the Gaussian bandpass filter. After that, the Scale and Curvature Invariant Ridge Detector (SCIRD) [17] is employed to detect the vessel map. SCIRD is simultaneously rotation, scale and curvature invariant, and removes the assumption of locally straight tubular structures based on the curved-support Gaussian model. After the vessel map is obtained, we compute the vessel density as the percentage of vessel pixels in respective region.

Following that, comparison of the pixels density between corresponding segmented layers and quadrants of images 1 and 2 from the same subject was done to evaluate the repeatability and variability of the OCTA system.

### Statistical analysis

Data analysis was carried out using IBM SPSS Statistics (version 22, IBM Corp, New York, USA) based on right and left eyes separately. A *p*-value of less than 0.05 is considered to be of statistical significance. Subject demographics and clinical characteristics were summarised as mean with standard deviation (SD) for age and IOP, and counts with percentages for gender, race, visual acuity and presence of cataract. Linear regression models were used to adjust for possible confounding effects of demographics and presence of cataract.

The measurements of vessel pixel density of the image 1 of each eyes were used to correlate with the subject demographics and clinical characteristics. Differences of measurements of each segmented layers were compared between gender and between presence of cataract using independent samples t-test. Pearson correlation coefficient (with scatter plots) were calculated to check for any linear relationship between layer measurements and age. Comparisons were done separately for right and left eyes.

The main outcome measure is the repeatability of the OCT angiographic vessels density in each segments and quadrants. This is presented as the mean difference of the vessels pixel density between first and repeated scans in each segments and quadrants. For this purpose, within-subject standard deviation (Sw), coefficient of variation (CV = Sw /overall mean), repeatability (1.96*√2*Sw), and their corresponding 95% confidence interval (95% C.I) were calculated. Paired t-test was used to compare if mean differences between 2 scans were of statistical significance.

## Results

### Demographics and clinical charateristics

A total of 176 eyes from 88 consecutive normal subjects were recruited in this study (Table 1). The subjects consists of 75 Chinese, 8 Malay, 4 Indian and 1 other race. The mean age of the subjects was 43.7 +/− 13.1 years with a range from 21 to 68 years old. There were 27 males and 61 females. In 47 subjects, only the optic nerve head was scanned. In 41 subjects both the optic nerve head and macular region were scanned. Subsets of each group had 2 OCTA scans for studying repeatability.

**Table 1:** Demography and clinical characteristics

	Total subject (*n* = 88)			
Demographics				
Age (years), mean ± SD	43.7	± 13.1	(range 21–68)	
Gender, n (%)				
Male	27	(30.7)		
Female	61	(69.3)		
Race, n (%)				
Chinese	75	(85.2)		
Malay	8	(9.1)		
Indian	4	(4.5)		
Others	1	(1.1)		
Clinical characteristics				
Presence of cataract, n (%)				
Clear lens	74	(84.1)		
Unilateral cataract	2	(2.3)		
Bilateral cataract	12	(13.6)		
Visual acuity, n (%)	Right		Left	
6/6	60	(68.2%)	64	(72.7%)
6/7.5	17	(19.3%)	15	(17.0%)
6/9	9	(10.2%)	8	(9.1%)
6/12	2	(2.3%)	1	(1.1%)
IOP (mmHg), mean ± SD	13.7	± 2.8	14.0	± 2.5

The unaided visual acuity were 6/12 or better in all eyes. The mean IOP was 13.7 +/− 2.8 mmHg in the right eyes and 14.0 +/− 2.5 mmHg in the left eyes. Twelve subjects were found to have mild cataract in bilateral eyes; 2 had mild cataract in either right or left eye; and 74 had normal clear lens. In our sample, presence of cataract was found only in subjects more than 45 years old.

### Image segmentation and subdivision

**Fig. 2:**
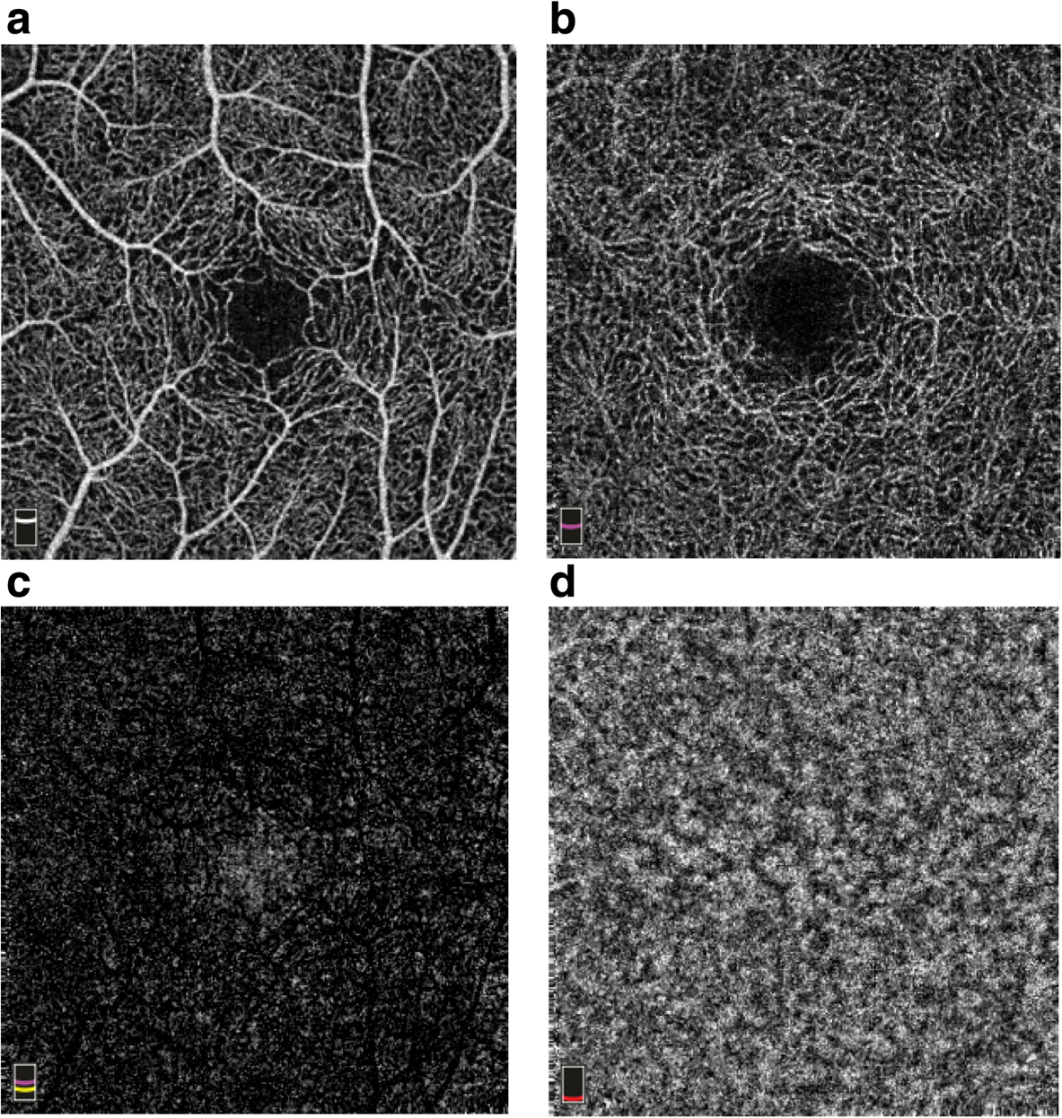
Macular image segmentation. **a** Superficial retina capillary plexus. **b** Deep retina capillary plexus. **c** Outer retina capillary plexus. **d** choriocapillaries

**Fig. 3:**
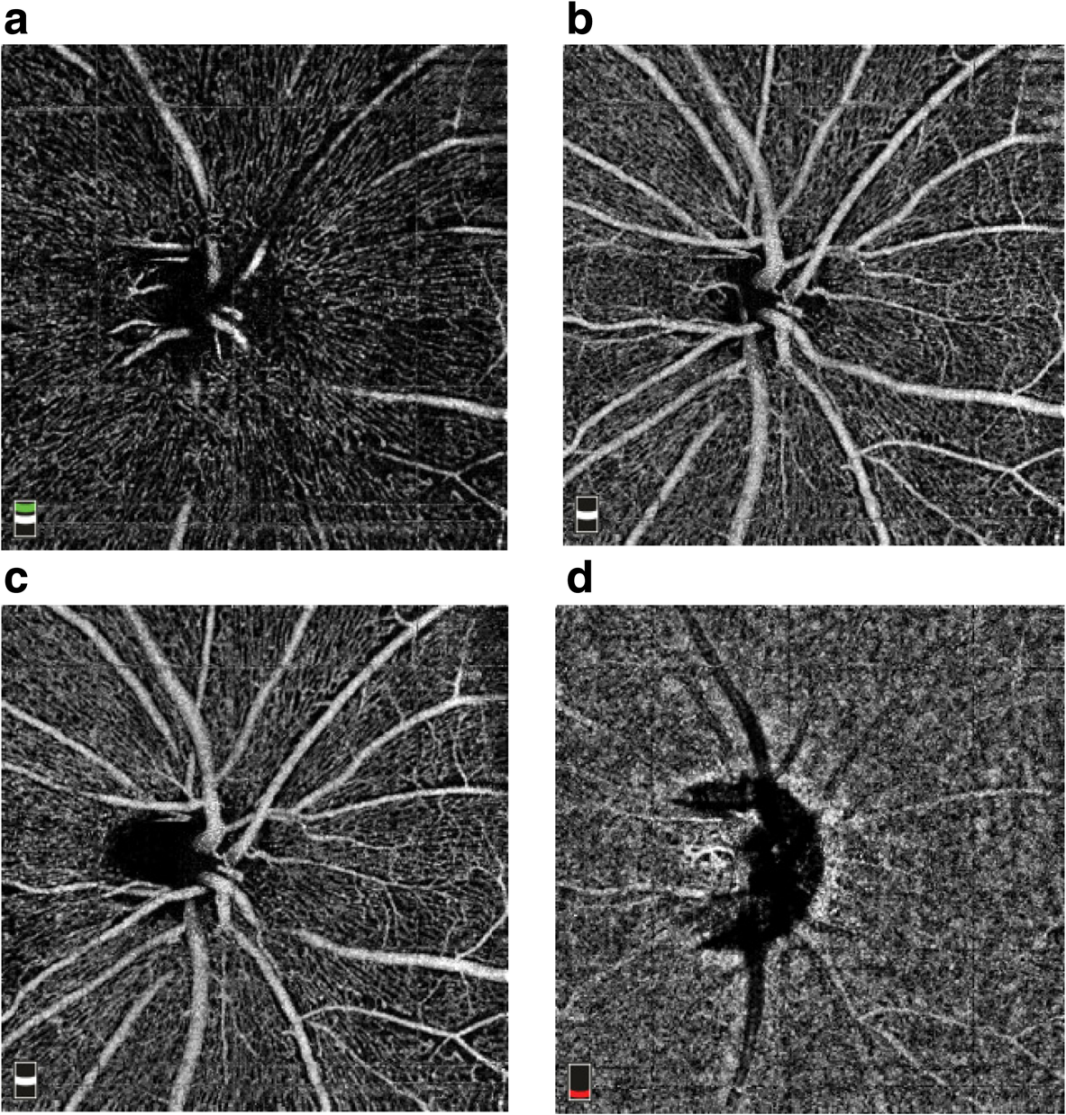
Optic disc image segmentation. **a** Vitreoretinal interface. **b** Superfical Optic nerve head. **c** Radial peripapillary capillaries. **d** Optic nerve at the level of the choroid

The OCTA system automatically segmented macular and optic disc images into 4 segments. Each segment was subdivided into 4 quadrants as described earlier (Figs. 2a-d and 3a-d).

### Vessel pixel density measurement

Vessel pixel density was calculated for each segmented layers (labelled as ‘segment’) and the 4 quadrants. For macular scan segments of the right and left eyes (Table 2), deep retina segment had the highest mean vessel pixel density (OD 0.239, OS 0.230) followed by choriocapillaries (OD 0.237, OS 0.215), outer retina (OD 0.179, OS 0.164) and superficial retina (OD 0.113, OS 0.111). In optic disc scan (Table 3), optic disc at choroid level was found to have highest mean vessel pixel density (OD 0.227, OS 0.236) followed by superficial nerve head (OD 0.216, OS 0.209), RPC (OD 0.140, OS 0.138) and optic disc at vitreoretinal interface (OD 0.084, OS 0.085).

**Table 2:** Vessel pixel density of macular for measurements of first scan

	Right		Left	
	Mean	(SD)	Mean	(SD)
Superficial Retina				
(OD = 40, OS = 41)				
Inferior	0.126	(0.017)	0.124	(0.020)
Superior	0.121	(0.017)	0.118	(0.025)
Nasal	0.105	(0.067)	0.120	(0.066)
Temporal	0.145	(0.047)	0.110	(0.055)
Segment	0.113	(0.014)	0.111	(0.016)
Deep Retina				
(OD = 40, OS = 41)				
Inferior	0.243	(0.039)	0.228	(0.056)
Superior	0.229	(0.042)	0.218	(0.055)
Nasal	0.221	(0.049)	0.248	(0.066)
Temporal	0.262	(0.134)	0.250	(0.125)
Segment	0.239	(0.040)	0.230	(0.053)
Outer Retina				
(OD = 40, OS = 41)				
Inferior	0.121	(0.087)	0.149	(0.084)
Superior	0.113	(0.089)	0.121	(0.089)
Nasal	0.215	(0.134)	0.195	(0.124)
Temporal	0.189	(0.107)	0.171	(0.092)
Segment	0.179	(0.061)	0.164	(0.071)
Choriocapillaries				
(OD = 40, OS = 41)				
Inferior	0.254	(0.049)	0.219	(0.064)
Superior	0.229	(0.068)	0.214	(0.077)
Nasal	0.218	(0.084)	0.214	(0.091)
Temporal	0.244	(0.085)	0.226	(0.058)
Segment	0.237	(0.059)	0.215	(0.066)

**Table 3:** Vessel pixel density of optic disc for measurements of first scan

	Right		Left	
	Mean	(SD)	Mean	(SD)
Vitreoretinal interface				
(OD = 83, OS = 81)				
Inferior	0.097	(0.087)	0.106	(0.075)
Superior	0.074	(0.061)	0.066	(0.068)
Nasal	0.076	(0.070)	0.077	(0.070)
Temporal	0.081	(0.072)	0.083	(0.076)
Segment	0.084	(0.051)	0.085	(0.048)
Superfical Nerve Head				
(OD = 86, OS = 85)				
Inferior	0.242	(0.074)	0.238	(0.083)
Superior	0.232	(0.077)	0.218	(0.076)
Nasal	0.232	(0.063)	0.207	(0.084)
Temporal	0.171	(0.082)	0.157	(0.090)
Segment	0.216	(0.048)	0.209	(0.050)
RPC				
(OD = 84, OS = 84)				
Inferior	0.184	(0.075)	0.186	(0.068)
Superior	0.133	(0.076)	0.124	(0.067)
Nasal	0.136	(0.079)	0.136	(0.082)
Temporal	0.100	(0.076)	0.093	(0.077)
Segment	0.140	(0.053)	0.138	(0.043)
Disc at choroid level				
(OD = 86, OS = 85)				
Inferior	0.228	(0.100)	0.239	(0.085)
Superior	0.257	(0.113)	0.266	(0.103)
Nasal	0.234	(0.102)	0.233	(0.102)
Temporal	0.191	(0.112)	0.199	(0.106)
Segment	0.227	(0.079)	0.236	(0.070)

In macular scan, denser vessel network were found in nasal quadrant in the outer retinal segment but temporal quadrant in the deep retinal segment for both eyes. In superficial retina and choriocapillaries segments, the quadrant with the highest vessel density differed between eyes. For example in the superficial retina, it was highest in temporal quadrant in the right eye (0.145), but inferior quadrant in the left eye (0.124). In choriocapillaries segment, vessel density was highest in inferior quadrant of the right eye (0.254), but temporal quadrant in the left eye (0.226).

On the other hand, with the exception of the optic disc scan at the level of the choroid, higher vessel density was found in the inferior quadrant of other optic nerve segments (Table 3). This pattern of distribution was evidenced in optic nerve scan at the level of vitreoretinal interface (OD 0.097, OS 0.106), radial peripapillary capillary (OD 0.184; OS 0.186) and superficial optic nerve head segments (OD O.242, OS 0.238). Optic nerve segment at the level of the choroid, in contrast, showed a higher vessel density in the superior quadrant (OD 0.257; OS 0.266).

We examined the association of age, presence of cataract and gender with the vessel pixel density measurements of each segmented layers in macular and optic disc, obtained during the first scan. No statistically significant positive or negative correlation between age and the segmented layer measurements for either eye were found (samples independent test; all *p* > 0.05). Neither were there statistically significant differences between presence of cataract and the segmented layer measurements for either eye (all *p* > 0.05). Interestingly, statistically significant differences were found between gender for left eye disc vitreous measurement (mean difference 0.024, 95% C.I 0.001 to 0.047, *p* = 0.041). Using linear regression models, our analysis showed that males had higher disc vitreous measurement compared to females for left eyes after controlling for age and presence of cataract (univariate analysis: 0.023, 95% C.I 0.001 to 0.046, *p* = 0.041; multivariable analysis: 0.023, 95% C.I 0 to 0.046, *p* = 0.051). The rest of the models did not show any factors to be of statistical significance.

### Measurement repeatability

The next section of the analysis was concerned with the repeatability of OCTA. Two repeated scan of good quality is essential for this purpose. Of the 88 subjects, a subset of 41 subjects had only 1 scan done for each eyes so they were automatically excluded from repeatability analysis. This resulted in 47 subjects with two scans eligible for repeatability tests. From the 47 subjects, only those with good quality scans were included for further evaluation. The vessel pixel measurement for the segments with significant motion artifacts were excluded from analysis. We did not discard the whole scan if certain segments were of good quality.

The results of the repeatability tests are shown in Additional file 1: Table S4; Additional file 2: Table S5; Additional file 3: Table S6 and Additional file 4: Table S7. The Additional file 1: Table S4 and Additional file 2: Table S5 illustrated the mean difference between first and second scan of different segments. The mean difference ranged from 0 to 0.022 in macular scan and 0 to 0.030 in optic disc scan. Using paired T-test, the mean differences did not reach significant level with the exception of deep retina segment in macular scan and temporal quadrant of superficial nerve head in optic disc scan of the right eye.

Further analysis revealed that within subject standard deviation of macular scans ranged from 0.008 to 0.076 and 0.006 to 0.098; the CV ranged from 6.4 to 31.1% and 5.3 to 59.4%; and the repeatability coefficient ranged from 0.022 to 0.211 and 0.017 to 0.272 for right and left eyes respectively (See Additional file 3).

Likewise, we found that within subject standard deviation of optic disc scans ranged from 0.027 to 0.078 and 0.028 to 0.066; the CV ranged from 14.3 to 77.4% and 13.5 to 75.3%; and repeatability coefficient ranged from 0.075 to 0.217 and 0.076 to 0.183 for right and left eyes respectively (See Additional file 4).

Our results indicate that segment vessel pixel density measurements generally showed better repeatability as compared to quadrant measurements. The only exception was found in the superior quadrant of superficial retina of the right eye, which showed better measurement repeatability than its segment and other quadrants. In macular scan, higher CV was found in either nasal and temporal quadrants of the segments for both right and left eye. While in optic disc scans, higher CV was found in temporal quadrants in most segments.

## Discussion

In recent years, there has been an increasing interest in OCTA as an investigative instrument to study vascular abnormalities in posterior segment eye conditions [1, 2, 7, 9]. OCTA, with en face segmentation technology, provides valuable information with regard to the vessels which supply the macula [18, 19] and optic disc [8, 20]. However, despite its potential usefulness, much uncertainty still exists about the reliability of this relatively new instrument in clinical application. In the present study, we use OCTA to determine the vessel density in each segment of the macula and optic disc. Moreover, we report the repeatability of these OCTA vessel density measurements.

Our results show that dense vascular networks are visible in the macular scan of normal subjects. The highest vessel density among all segments of the macula is found in the deep retina layer (OD 0.239; OS 0.230). The vessel density in this layer is slightly more than the underlying choroidal segment (OD 0.237; OS 0.215). In contrast, the superficial retinal layer has the lowest vessels density (OD 0.113; OS 0.111). These findings indicate that higher blood perfusion is found in deeper than superficial layers of the macular in normal subjects. These results are in line with those of a previous study [21].

In optic disc scans, we also observed dense vessel networks located around and in the optic disc. Analysis from the segmented image of the optic disc showed that highest vessel density was found at the segment located at the level of the choroid(OD 0.227; OS 0.236), followed by nerve head layer (OD 0.216, OS 0.209). It was somewhat surprising that RPC (OD 0.140, OS 0.138) - capillary network potentially associated with glaucoma, contained lower vessel density than choroid and nerve head layer. The vessel density found in this layer is also lower than that of previous reported level (0.21 +/− 0.053) [20]. An explanation for the relatively lower RPC density in the present study as compared with previous report might be that our subjects are of older age. It had been demonstrated that vessels density decreased with age annually [18].

RPC networks are important in nourishing peripapillary RNFL due to its unique location and distribution [22]. Reduction of flow in RPC due to pathological condition will have detrimental effects to the retinal nerve fiber layer and optic disc [10]. Our data, on the other hand, demonstrated dense microvessels in ONH and peripapillary choroid in healthy subjects which suggested potentially important role of microvessels in these segments in maintaining the integrity of optic nerve head, in additional to RPC networks. This inference is further supported by previous studies which demonstrated reduction of microvessels in ONH [12] and peripapillary choroid [23] in glaucomatous optic neuropathy.

In the present study, in additional to assessment of segment images, we further divided all segments of macular and optic disc scans into 4 quadrants in order to demonstrate the pattern of vessel distribution in the quadrants of every segments. To the best of our knowledge, this has not been previously studied. We found that, in macular scans, there was greater variability in the quadrant with highest vessel density depending on segment and laterality of the eyes. The lack of consistency suggested that there may be no specific preferential quadrant distribution of vessels in the macular. In contrast, quadrant distribution of vessel in optic disc segments was more consistent. A higher vessels density was found in inferior and superior quadrant. In normal subjects, it is known that healthy RNFL and the neuro-retinal rim are thickest in inferior and superior quadrants of optic disc [24]. Denser distribution of vessels to these 2 quadrants suggested a positive association between rim thickness and blood perfusion. This is in agreement with Yu’s finding which further showed correlation between RPC and RNFL thickness [22].

Another aim of the present study is to evaluate the OCT angiography test-retest repeatability. We found that macular OCT angiography scan showed a range of measurement variability. The mean difference between first and second scan were not statistically significant with the exception of deep retina segment for the right eye. The CV of all macular segments was 5.3–17.2%, and the repeatability coefficient was 0.017–0.082, indicating good repeatability of macular OCT angiography. However, lower test-retest repeatability was found when the macular en face segments were divided into smaller quadrants (CV 6.4–59.4%; repeatability coefficient 0.022–0.272). Of the 4 quadrants, nasal and temporal quadrants of macular OCT angiography showed the highest variability in the present study. Our findings suggested that segment image is more repeatable and consistent than quadrant image in macular OCT angiography. Based on our repeatability and variability results, it seems that segmented image analysis in OCTA may have more clinical use in follow up scanning, and intepretation of changes in vessel density in quadrants may be difficult at this juncture. Interestingly, in an earlier study to determine the reproducibility of OCT measurement of normal human peripapillary RNFL thickness, Jones et al. reported that the mean coefficient of variation increases with subdivision of measurement area [25]. Likewise, their CV was also higher in nasal quadrant than inferior and superior quadrants. Our findings are in agreement with their observation.

Comparatively, optic disc OCT angiography demonstrated wider range of measurement variability. The mean difference between first and second scan were not statistically significant with the exception of temporal quadrant of superficial nerve head for the right eye. The CV of the optic disc segments range from 13.5 to 29.6%, and repeatability coefficient was 0.075–0.145, indicating a lower test-retest repeatability of optic disc scan than macular. Similar to macular scan, en face segments of optic disc demonstrated better repeatability than quadrant images (CV 13.5–29.6% vs 16.9–77.4%). Image with highest variability was found in temporal and nasal quadrants in most optic disc segments. This result seems to be consistent with those of Jia et al., which reported that the flow index and vessels density in whole disc measurements had lower measurement variability than the smaller regional disc measurement [8].

The repeatability of OCT angiography of optic disc vessels density has been described previously. In a sample of 12 normal eyes, Liu et al. reported within-visit repeatability CVs of 1.9% for peripapillary vessel density [7]. Based on a sample of 3 healthy subjects, Wang et al. also reported low CVs of 1.03% for optic disc vessel density and flow index [9]. Similarly, in 2 separate studies involving 3 and 4 normal subjects, Jia et al. reported low intra-visit CVs of 6.2 and 1.2% respectively [8, 15]. Overall, earlier studies demonstrated good repeatability of OCT angiography of the optic disc. It is important to note, however, that the studies were conducted in the same institution with small sample size. Using a larger sample size, our study was not able to reproduce low CVs to suggest good repeatability of OCT angiography of the disc.

Our findings have clinical significance in using OCT angiography as an instrument to monitor optic disc perfusion. While studies have demonstrated that OCTA is useful for assessment of optic disc perfusion [8, 9, 11, 15], caution should be exercised when interpreting serial optic disc OCTA images due to potential test-retest variability of the measurements. The information from optic disc OCTA may provide support for clinical suspicion of, rather than conclusive evidence of true microvasculature changes.

Our study is not without its limitations. In this study, we recruited subjects with BCVA of 6/12 or better to represent healthy subject. Presence of mild cataract was found in 14 subjects (13.6%) and this could have affected the quality of the images and pixel density measurement. The mean age of 43.7 years of our study subjects may not be an ideal representation of elderly population in whom retinal and ONH diseases are more commonly diagnosed. Advancing age is a major risk factor for some common choroidal, retinal and ONH diseases associated with microvasculature changes, notably AMD [10, 26] and POAG [27, 28]. In this clinical setting, other age-related eye conditions such as cataract and ocular surface disease frequently occur simultaneously in elderly subjects [29] and will inevitably influence the reliability of the angiographic result. Additionally, the axial length was not measured during subject recruitment. Axial length potentially influences image quality of OCT and therefore should be taken into consideration in similar study in future. It is known that central deviation of OCTA image during acquisition potentially affects the measurement repeatability. To address this concern, we identified the center and border of disc image and used it as a guide to partition the segment into 4 quadrants. However, we acknowledge that this extra step was not done during macular images partitioning process. In additional to that, we did not include peripapillary area in deciding the border of optic nerve head. A larger border of optic nerve head to include the peripapillary area will provide additional information about peripapillary capillaries. In term of result intepretation, a note of caution is due here since we did not include the standard deviation as a criteria in image selection. As CV is an indicator of standard deviation (CV = Sw /overall mean), we found a relatively large CV range in the repeatability analysis. It should be noted that we investigated only one specific OCTA model in the present study, the result may not represent the different commercially available OCTA. In a study comparing 7 different OCT, Pierro et al. reported that RNFL thickness measurement variability was found to be different among the instruments [30]. Given that OCTA relies on OCT technology as the system platform, we speculate the reliability of OCTA may also differ among the different systems. Further study is needed for more conclusive answers. On the other hand, as mentioned in previous section, we used Optovue version 2016.2.0.35 in OCTA image acquisition in the study. While the vessel was succesfully demonstrated, this earlier OCTA model did not feature the ability to quatify the microvasculature density. Our novel in house software was developed to overcome the shortcoming. To our knowledge, latest optovue model comes with new feature allowing microvessel quantification to be done using built-in software. It will be interesting to compare the the two software in future study.

## Conclusions

The OCTA system effectively demonstrated dense microvascular network at macular and optic disc. The study found that the OCTA of macula in healthy subjects showed a good test-retest repeatability that is comparable to previous studies. However, a significant measurement variability was found on optic disc OCTA. Additionally, a lower measurement repeatability was found when the en face segments of macular and optic disc are divided into smaller quadrants. Clinical Interpretation of OCTA should take into account the test-retest repeatability of the system for a more meaningful interpretation.

## Additional files

Additional file 1: Comparisons of first and second scans of macular OCTA. (DOC 67 kb)

Additional file 2: Comparisons of first and second scans of optic disc OCTA. (DOC 68 kb)

Additional file 3: Repeatability tests of macular OCTA. (DOC 69 kb)

Additional file 4: Repeatability tests of optic disc OCTA. (DOC 68 kb)

## Abbreviations

AMD: Age-related macular degeneration
BCVA: Best corrected visual acuity
CV: Coefficient of variation
FA: Fluoroscein angiography
ICG: Indocyanine green angiography
IOP: Intraocular pressure
OCT: Optical coherence tomography
OCTA: Optical coherence tomography angiography
OD: Right eye
ONH: Optic nerve head
OS: Left eye
POAG: Primary open angle glaucoma
RNFL: Retinal nerve fiber layer
RPC: Radial peripapillary capillaries
SCIRD: Scale and curvature invariant ridge detector
SD: Standard deviation
SSADA: Split-spectrum amplitude-decorrelation angiography
SW: Within subject standard deviation
