# Supplementary material for: Optical coherence tomography angiography of the macula and optic nerve head: microvascular density and test-retest repeatability in normal subjects
Source: BMC Ophthalmol. 2018 Dec 10;18:315. doi: 10.1186/s12886-018-0976-y (PMC6288920; doi:10.1186/s12886-018-0976-y)
Supplement: Supplementary file 1 — Comparisons of first and second scans of macular OCTA. (DOC 67 kb) [file 12886_2018_976_MOESM1_ESM.doc]

Table S4: Comparisons of first and second scans of macular OCTA

| **Right Eye** | | | | | | | |  | **Left Eye** | | | | | | | |
| --- | --- | --- | --- | --- | --- | --- | --- | --- | --- | --- | --- | --- | --- | --- | --- | --- |
|  | First Scan | | Second Scan | | Difference (Scan 2 - Scan 1) | | |  |  | First Scan | | Second Scan | | Difference (Scan 2 - Scan 1) | | |
|  | Mean | (SD) | Mean | (SD) | Mean | 95% C.I | P value |  |  | Mean | (SD) | Mean | (SD) | Mean | 95% C.I | P value |
| Superficial Retina |  |  |  |  |  |  |  |  | Superficial Retina |  |  |  |  |  |  |  |
| (OD =30) |  |  |  |  |  |  |  |  | (OS=30) |  |  |  |  |  |  |  |
| Inferior | 0.126 | (0.018) | 0.128 | (0.022) | 0.002 | (-0.007 to 0.011) | 0.633 |  | Inferior | 0.128 | (0.021) | 0.127 | (0.020) | -0.001 | (-0.006 to 0.003) | 0.598 |
| Superior | 0.121 | (0.018) | 0.125 | (0.018) | 0.003 | (-0.001 to 0.007) | 0.116 |  | Superior | 0.125 | (0.018) | 0.128 | (0.020) | 0.002 | (-0.003 to 0.007) | 0.391 |
| Nasal | 0.114 | (0.074) | 0.125 | (0.075) | 0.011 | (-0.008 to 0.031) | 0.244 |  | Nasal | 0.130 | (0.074) | 0.120 | (0.077) | -0.010 | (-0.049 to 0.030) | 0.625 |
| Temporal | 0.140 | (0.042) | 0.138 | (0.046) | -0.002 | (-0.014 to 0.01) | 0.696 |  | Temporal | 0.103 | (0.027) | 0.117 | (0.056) | 0.014 | (-0.007 to 0.034) | 0.181 |
| Total | 0.117 | (0.014) | 0.123 | (0.027) | 0.006 | (-0.005 to 0.017) | 0.262 |  | Total | 0.116 | (0.013) | 0.115 | (0.014) | -0.002 | (-0.005 to 0.002) | 0.333 |
| Deep Retina |  |  |  |  |  |  |  |  | Deep Retina |  |  |  |  |  |  |  |
| (OD=30) |  |  |  |  |  |  |  |  | (OS=30) |  |  |  |  |  |  |  |
| Inferior | 0.247 | (0.035) | 0.225 | (0.037) | -0.021 | (-0.032 to -0.01) | < 0.001 |  | Inferior | 0.242 | (0.045) | 0.245 | (0.038) | 0.003 | (-0.009 to 0.015) | 0.604 |
| Superior | 0.239 | (0.040) | 0.232 | (0.056) | -0.007 | (-0.022 to 0.007) | 0.315 |  | Superior | 0.236 | (0.033) | 0.232 | (0.040) | -0.004 | (-0.018 to 0.010) | 0.533 |
| Nasal | 0.233 | (0.048) | 0.238 | (0.056) | 0.005 | (-0.007 to 0.017) | 0.402 |  | Nasal | 0.274 | (0.043) | 0.264 | (0.055) | -0.010 | (-0.026 to 0.005) | 0.170 |
| Temporal | 0.279 | (0.108) | 0.289 | (0.088) | 0.010 | (-0.031 to 0.051) | 0.619 |  | Temporal | 0.263 | (0.090) | 0.240 | (0.090) | -0.022 | (-0.074 to 0.030) | 0.388 |
| Total | 0.251 | (0.036) | 0.241 | (0.043) | -0.011 | (-0.019 to -0.002) | 0.020 |  | Total | 0.249 | (0.034) | 0.247 | (0.038) | -0.003 | (-0.010 to 0.005) | 0.443 |
| Outer Retina |  |  |  |  |  |  |  |  | Outer Retina |  |  |  |  |  |  |  |
| (OD=30) |  |  |  |  |  |  |  |  | (OD=30) |  |  |  |  |  |  |  |
| Inferior | 0.146 | (0.078) | 0.149 | (0.079) | 0.003 | (-0.017 to 0.023) | 0.786 |  | Inferior | 0.174 | (0.073) | 0.166 | (0.074) | -0.008 | (-0.026 to 0.010) | 0.351 |
| Superior | 0.143 | (0.082) | 0.146 | (0.092) | 0.003 | (-0.017 to 0.023) | 0.740 |  | Superior | 0.146 | (0.086) | 0.139 | (0.077) | -0.007 | (-0.028 to 0.014) | 0.484 |
| Nasal | 0.200 | (0.105) | 0.193 | (0.110) | -0.008 | (-0.038 to 0.022) | 0.606 |  | Nasal | 0.197 | (0.094) | 0.209 | (0.083) | 0.013 | (-0.018 to 0.044) | 0.407 |
| Temporal | 0.209 | (0.094) | 0.222 | (0.091) | 0.013 | (-0.012 to 0.038) | 0.304 |  | Temporal | 0.189 | (0.078) | 0.168 | (0.076) | -0.020 | (-0.048 to 0.007) | 0.134 |
| Total | 0.187 | (0.050) | 0.187 | (0.058) | 0.000 | (-0.014 to 0.014) | 0.975 |  | Total | 0.176 | (0.064) | 0.178 | (0.050) | 0.003 | (-0.013 to 0.019) | 0.723 |
| Retinal Choroid |  |  |  |  |  |  |  |  | Retinal Choroid |  |  |  |  |  |  |  |
| (OD=30) |  |  |  |  |  |  |  |  | (OS=30) |  |  |  |  |  |  |  |
| Inferior | 0.268 | (0.039) | 0.258 | (0.046) | -0.010 | (-0.026 to 0.006) | 0.218 |  | Inferior | 0.238 | (0.056) | 0.244 | (0.051) | 0.007 | (-0.006 to 0.020) | 0.287 |
| Superior | 0.247 | (0.058) | 0.250 | (0.047) | 0.003 | (-0.012 to 0.018) | 0.666 |  | Superior | 0.245 | (0.053) | 0.251 | (0.056) | 0.006 | (-0.009 to 0.022) | 0.400 |
| Nasal | 0.239 | (0.071) | 0.242 | (0.074) | 0.003 | (-0.015 to 0.022) | 0.720 |  | Nasal | 0.240 | (0.079) | 0.247 | (0.087) | 0.007 | (-0.007 to 0.022) | 0.319 |
| Temporal | 0.270 | (0.072) | 0.270 | (0.058) | 0.000 | (-0.032 to 0.032) | 0.986 |  | Temporal | 0.242 | (0.052) | 0.238 | (0.055) | -0.004 | (-0.026 to 0.017) | 0.682 |
| Total | 0.258 | (0.045) | 0.257 | (0.042) | -0.001 | (-0.012 to 0.010) | 0.852 |  | Total | 0.242 | (0.045) | 0.249 | (0.047) | 0.007 | (-0.002 to 0.016) | 0.116 |

SD: Standard Deviation; C.I: Confidence Interval
