# Supplementary material for: Optical coherence tomography angiography of the macula and optic nerve head: microvascular density and test-retest repeatability in normal subjects
Source: BMC Ophthalmol. 2018 Dec 10;18:315. doi: 10.1186/s12886-018-0976-y (PMC6288920; doi:10.1186/s12886-018-0976-y)
Supplement: Supplementary file 2 — Comparisons of first and second scans of optic disc OCTA. (DOC 68 kb) [file 12886_2018_976_MOESM2_ESM.doc]

Table S5: Comparisons of first and second scans of optic disc OCTA

| **Right Eye** | | | | | | | |  | **Left Eye** | | | | | | | |
| --- | --- | --- | --- | --- | --- | --- | --- | --- | --- | --- | --- | --- | --- | --- | --- | --- |
|  | First Scan | | Second Scan | | Difference (Scan 2 - Scan 1) | | |  |  | First Scan | | Second Scan | | Difference (Scan 2 - Scan 1) | | |
|  | Mean | (SD) | Mean | (SD) | Mean | 95% C.I | P value |  |  | Mean | (SD) | Mean | (SD) | Mean | 95% C.I | P value |
| Disc Vitreous |  |  |  |  |  |  |  |  | Disc Vitreous |  |  |  |  |  |  |  |
| (OD = 40) |  |  |  |  |  |  |  |  | (OS = 40) |  |  |  |  |  |  |  |
| Inferior | 0.105 | (0.099) | 0.112 | (0.093) | 0.008 | (-0.013 to 0.028) | 0.460 |  | Inferior | 0.125 | (0.083) | 0.117 | (0.086) | -0.009 | (-0.025 to 0.008) | 0.316 |
| Superior | 0.071 | (0.061) | 0.090 | (0.090) | 0.019 | (-0.004 to 0.043) | 0.109 |  | Superior | 0.082 | (0.078) | 0.065 | (0.082) | -0.017 | (-0.033 to 0.000) | 0.052 |
| Nasal | 0.086 | (0.076) | 0.079 | (0.076) | -0.007 | (-0.025 to 0.011) | 0.414 |  | Nasal | 0.082 | (0.073) | 0.087 | (0.079) | 0.005 | (-0.014 to 0.024) | 0.567 |
| Temporal | 0.080 | (0.075) | 0.094 | (0.087) | 0.014 | (-0.012 to 0.039) | 0.289 |  | Temporal | 0.100 | (0.083) | 0.095 | (0.076) | -0.005 | (-0.031 to 0.021) | 0.718 |
| Total | 0.088 | (0.055) | 0.097 | (0.058) | 0.009 | (-0.004 to 0.021) | 0.158 |  | Total | 0.101 | (0.052) | 0.092 | (0.054) | -0.009 | (-0.022 to 0.003) | 0.145 |
| Disc RPC |  |  |  |  |  |  |  |  | Disc RPC |  |  |  |  |  |  |  |
| (OD = 41) |  |  |  |  |  |  |  |  | (OS = 43) |  |  |  |  |  |  |  |
| Inferior | 0.178 | (0.081) | 0.173 | (0.093) | -0.004 | (-0.029 to 0.021) | 0.733 |  | Inferior | 0.194 | (0.074) | 0.184 | (0.079) | -0.010 | (-0.030 to 0.011) | 0.362 |
| Superior | 0.128 | (0.086) | 0.129 | (0.094) | 0.000 | (-0.022 to 0.023) | 0.969 |  | Superior | 0.112 | (0.061) | 0.105 | (0.063) | -0.007 | (-0.022 to 0.009) | 0.384 |
| Nasal | 0.137 | (0.087) | 0.150 | (0.082) | 0.013 | (-0.007 to 0.033) | 0.207 |  | Nasal | 0.155 | (0.093) | 0.154 | (0.092) | 0.000 | (-0.025 to 0.025) | 0.976 |
| Temporal | 0.065 | (0.070) | 0.068 | (0.082) | 0.003 | (-0.021 to 0.026) | 0.821 |  | Temporal | 0.073 | (0.075) | 0.084 | (0.070) | 0.011 | (-0.015 to 0.037) | 0.395 |
| Total | 0.129 | (0.058) | 0.132 | (0.052) | 0.004 | (-0.011 to 0.018) | 0.636 |  | Total | 0.135 | (0.039) | 0.131 | (0.044) | -0.003 | (-0.016 to 0.009) | 0.587 |
| Nerve Head |  |  |  |  |  |  |  |  | Nerve Head |  |  |  |  |  |  |  |
| (OD = 41) |  |  |  |  |  |  |  |  | (OS = 43) |  |  |  |  |  |  |  |
| Inferior | 0.225 | (0.065) | 0.229 | (0.069) | 0.004 | (-0.013 to 0.022) | 0.603 |  | Inferior | 0.229 | (0.088) | 0.228 | (0.078) | -0.001 | (-0.019 to 0.017) | 0.890 |
| Superior | 0.210 | (0.064) | 0.217 | (0.081) | 0.007 | (-0.016 to 0.030) | 0.550 |  | Superior | 0.195 | (0.076) | 0.196 | (0.073) | 0.001 | (-0.015 to 0.016) | 0.926 |
| Nasal | 0.207 | (0.063) | 0.209 | (0.075) | 0.002 | (-0.022 to 0.026) | 0.852 |  | Nasal | 0.215 | (0.085) | 0.221 | (0.076) | 0.007 | (-0.014 to 0.027) | 0.521 |
| Temporal | 0.144 | (0.084) | 0.174 | (0.087) | 0.030 | (0.004 to 0.056) | 0.023 |  | Temporal | 0.151 | (0.098) | 0.151 | (0.086) | 0.000 | (-0.029 to 0.029) | 0.999 |
| Total | 0.198 | (0.041) | 0.204 | (0.045) | 0.006 | (-0.007 to 0.019) | 0.365 |  | Total | 0.195 | (0.049) | 0.199 | (0.045) | 0.004 | (-0.008 to 0.016) | 0.539 |
| Disc Choroid |  |  |  |  |  |  |  |  | Disc Choroid |  |  |  |  |  |  |  |
| (OD = 41) |  |  |  |  |  |  |  |  | (OS = 43) |  |  |  |  |  |  |  |
| Inferior | 0.235 | (0.103) | 0.231 | (0.099) | -0.004 | (-0.039 to 0.031) | 0.812 |  | Inferior | 0.261 | (0.090) | 0.261 | (0.090) | 0.000 | (-0.029 to 0.028) | 0.979 |
| Superior | 0.278 | (0.106) | 0.282 | (0.123) | 0.004 | (-0.028 to 0.037) | 0.788 |  | Superior | 0.297 | (0.097) | 0.286 | (0.089) | -0.011 | (-0.034 to 0.013) | 0.365 |
| Nasal | 0.239 | (0.111) | 0.232 | (0.112) | -0.008 | (-0.043 to 0.028) | 0.660 |  | Nasal | 0.260 | (0.099) | 0.248 | (0.116) | -0.012 | (-0.037 to 0.014) | 0.363 |
| Temporal | 0.226 | (0.116) | 0.246 | (0.114) | 0.020 | (-0.011 to 0.051) | 0.200 |  | Temporal | 0.243 | (0.099) | 0.219 | (0.103) | -0.024 | (-0.048 to 0.000) | 0.054 |
| Total | 0.249 | (0.074) | 0.249 | (0.085) | 0.000 | (-0.024 to 0.023) | 0.978 |  | Total | 0.264 | (0.067) | 0.259 | (0.060) | -0.005 | (-0.02 to 0.011) | 0.553 |

SD: Standard Deviation; C.I: Confidence Interval
