# Supplementary material for: Optical coherence tomography angiography of the macula and optic nerve head: microvascular density and test-retest repeatability in normal subjects
Source: BMC Ophthalmol. 2018 Dec 10;18:315. doi: 10.1186/s12886-018-0976-y (PMC6288920; doi:10.1186/s12886-018-0976-y)
Supplement: Supplementary file 3 — Repeatability tests of macular OCTA. (DOC 69 kb) [file 12886_2018_976_MOESM3_ESM.doc]

Table S6: Repeatability tests of macular OCTA

| **Right Eye** | | | | | | | |  | **Left Eye** | | | | | | | |
| --- | --- | --- | --- | --- | --- | --- | --- | --- | --- | --- | --- | --- | --- | --- | --- | --- |
|  |  | Overall mean | Sw | (95% C.I) | CV | Repeatability | (95% C.I) |  |  |  | Overall mean | Sw | (95% C.I) | CV | Repeatability | (95% C.I) |
| Superficial Retina | |  |  |  |  |  |  |  | Superficial Retina | |  |  |  |  |  |  |
| (OD = 30) | |  |  |  |  |  |  |  | (OS = 30) | |  |  |  |  |  |  |
|  | Inferior | 0.127 | 0.016 | (0.012 to 0.02) | 12.6% | 0.044 | (0.033 to 0.056) |  |  | Inferior | 0.127 | 0.009 | (0.006 to 0.011) | 6.8% | 0.024 | (0.018 to 0.03) |
|  | Superior | 0.123 | 0.008 | (0.006 to 0.01) | 6.4% | 0.022 | (0.016 to 0.027) |  |  | Superior | 0.126 | 0.009 | (0.007 to 0.012) | 7.4% | 0.026 | (0.019 to 0.033) |
|  | Nasal | 0.120 | 0.037 | (0.028 to 0.047) | 31.1% | 0.103 | (0.077 to 0.129) |  |  | Nasal | 0.125 | 0.074 | (0.056 to 0.093) | 59.4% | 0.206 | (0.154 to 0.258) |
|  | Temporal | 0.139 | 0.023 | (0.017 to 0.028) | 16.2% | 0.062 | (0.047 to 0.078) |  |  | Temporal | 0.110 | 0.040 | (0.03 to 0.05) | 36.0% | 0.110 | (0.082 to 0.138) |
|  | Segment | 0.120 | 0.021 | (0.015 to 0.026) | 17.2% | 0.057 | (0.043 to 0.072) |  |  | Segment | 0.115 | 0.006 | (0.005 to 0.008) | 5.3% | 0.017 | (0.013 to 0.021) |
| Deep Retina | |  |  |  |  |  |  |  | Deep Retina | |  |  |  |  |  |  |
| (OD = 30) | |  |  |  |  |  |  |  | (OS = 30) | |  |  |  |  |  |  |
|  | Inferior | 0.236 | 0.025 | (0.019 to 0.031) | 10.5% | 0.069 | (0.051 to 0.086) |  |  | Inferior | 0.244 | 0.023 | (0.017 to 0.029) | 9.4% | 0.064 | (0.048 to 0.08) |
|  | Superior | 0.235 | 0.028 | (0.021 to 0.035) | 11.9% | 0.078 | (0.058 to 0.097) |  |  | Superior | 0.234 | 0.026 | (0.019 to 0.033) | 11.1% | 0.072 | (0.054 to 0.09) |
|  | Nasal | 0.236 | 0.022 | (0.017 to 0.028) | 9.5% | 0.062 | (0.046 to 0.077) |  |  | Nasal | 0.269 | 0.029 | (0.022 to 0.037) | 10.9% | 0.081 | (0.06 to 0.101) |
|  | Temporal | 0.284 | 0.076 | (0.057 to 0.095) | 26.7% | 0.211 | (0.157 to 0.264) |  |  | Temporal | 0.251 | 0.098 | (0.073 to 0.123) | 39.1% | 0.272 | (0.203 to 0.341) |
|  | Segment | 0.246 | 0.018 | (0.013 to 0.023) | 7.3% | 0.050 | (0.037 to 0.063) |  |  | Segment | 0.248 | 0.014 | (0.01 to 0.017) | 5.6% | 0.038 | (0.029 to 0.048) |
| Outer Retina | |  |  |  |  |  |  |  | Outer Retina | |  |  |  |  |  |  |
| (OD = 30) | |  |  |  |  |  |  |  | (OS = 30) | |  |  |  |  |  |  |
|  | Inferior | 0.148 | 0.037 | (0.028 to 0.047) | 25.4% | 0.104 | (0.078 to 0.13) |  |  | Inferior | 0.170 | 0.034 | (0.025 to 0.042) | 19.8% | 0.093 | (0.07 to 0.117) |
|  | Superior | 0.144 | 0.037 | (0.028 to 0.046) | 25.6% | 0.102 | (0.076 to 0.128) |  |  | Superior | 0.143 | 0.040 | (0.03 to 0.05) | 27.8% | 0.110 | (0.082 to 0.138) |
|  | Nasal | 0.197 | 0.056 | (0.042 to 0.07) | 28.6% | 0.156 | (0.116 to 0.195) |  |  | Nasal | 0.203 | 0.058 | (0.044 to 0.073) | 28.7% | 0.162 | (0.121 to 0.202) |
|  | Temporal | 0.216 | 0.047 | (0.035 to 0.059) | 22.0% | 0.132 | (0.098 to 0.165) |  |  | Temporal | 0.179 | 0.053 | (0.039 to 0.066) | 29.4% | 0.146 | (0.109 to 0.182) |
|  | Segment | 0.187 | 0.026 | (0.019 to 0.033) | 13.9% | 0.072 | (0.054 to 0.091) |  |  | Segment | 0.177 | 0.030 | (0.022 to 0.037) | 16.7% | 0.082 | (0.061 to 0.103) |
| Choriocapillaries | |  |  |  |  |  |  |  | Choriocapillaries | |  |  |  |  |  |  |
| (OD = 30) | |  |  |  |  |  |  |  | (OS = 30) | |  |  |  |  |  |  |
|  | Inferior | 0.263 | 0.030 | (0.023 to 0.038) | 11.5% | 0.084 | (0.063 to 0.105) |  |  | Inferior | 0.241 | 0.024 | (0.018 to 0.03) | 10.1% | 0.067 | (0.05 to 0.084) |
|  | Superior | 0.249 | 0.027 | (0.021 to 0.034) | 11.0% | 0.076 | (0.057 to 0.095) |  |  | Superior | 0.248 | 0.029 | (0.021 to 0.036) | 11.6% | 0.080 | (0.06 to 0.1) |
|  | Nasal | 0.241 | 0.035 | (0.026 to 0.043) | 14.4% | 0.096 | (0.072 to 0.12) |  |  | Nasal | 0.244 | 0.027 | (0.02 to 0.034) | 11.2% | 0.076 | (0.056 to 0.095) |
|  | Temporal | 0.270 | 0.060 | (0.045 to 0.075) | 22.1% | 0.165 | (0.123 to 0.207) |  |  | Temporal | 0.240 | 0.040 | (0.03 to 0.05) | 16.7% | 0.111 | (0.083 to 0.14) |
|  | Segment | 0.257 | 0.021 | (0.016 to 0.026) | 8.2% | 0.059 | (0.044 to 0.073) |  |  | Segment | 0.245 | 0.018 | (0.013 to 0.022) | 7.3% | 0.049 | (0.037 to 0.062) |

Sw: Within-subject standard deviation; CV: Coefficient of variation is calculated as Sw / overall mean; Repeatability is calculated as 1.96*√2*Sw; C.I: Confidence Interval

Overall mean: The mean of the scan 1 & 2
