# Supplementary material for: Optical coherence tomography angiography of the macula and optic nerve head: microvascular density and test-retest repeatability in normal subjects
Source: BMC Ophthalmol. 2018 Dec 10;18:315. doi: 10.1186/s12886-018-0976-y (PMC6288920; doi:10.1186/s12886-018-0976-y)
Supplement: Supplementary file 4 — Repeatability tests of optic disc OCTA. (DOC 68 kb) [file 12886_2018_976_MOESM4_ESM.doc]

Table S7: Repeatability tests of optic disc OCTA

| **Right Eye** | | | | | | | |  | **Left Eye** | | | | | | | |
| --- | --- | --- | --- | --- | --- | --- | --- | --- | --- | --- | --- | --- | --- | --- | --- | --- |
|  |  | Overall mean | Sw | (95% C.I) | CV | Repeatability | (95% C.I) |  |  |  | Overall mean | Sw | (95% C.I) | CV | Repeatability | (95% C.I) |
| Vitreoretinal interface | | |  |  |  |  |  |  | Vitreoretinal interface | | |  |  |  |  |  |
| (OD = 40) | |  |  |  |  |  |  |  | (OS = 40) | |  |  |  |  |  |  |
|  | Inferior | 0.109 | 0.045 | (0.035 to 0.055) | 41.7% | 0.125 | (0.098 to 0.153) |  |  | Inferior | 0.121 | 0.038 | (0.029 to 0.046) | 31.0% | 0.104 | (0.081 to 0.127) |
|  | Superior | 0.081 | 0.053 | (0.041 to 0.065) | 65.9% | 0.147 | (0.115 to 0.179) |  |  | Superior | 0.073 | 0.038 | (0.03 to 0.047) | 52.4% | 0.107 | (0.083 to 0.13) |
|  | Nasal | 0.083 | 0.040 | (0.031 to 0.048) | 47.8% | 0.110 | (0.086 to 0.134) |  |  | Nasal | 0.085 | 0.042 | (0.032 to 0.051) | 49.0% | 0.115 | (0.09 to 0.14) |
|  | Temporal | 0.087 | 0.057 | (0.044 to 0.069) | 65.5% | 0.158 | (0.123 to 0.192) |  |  | Temporal | 0.098 | 0.057 | (0.045 to 0.07) | 58.4% | 0.158 | (0.124 to 0.193) |
|  | Segment | 0.092 | 0.027 | (0.021 to 0.033) | 29.5% | 0.075 | (0.059 to 0.092) |  |  | Segment | 0.096 | 0.029 | (0.022 to 0.035) | 29.6% | 0.079 | (0.062 to 0.097) |
| Nerve Head | |  |  |  |  |  |  |  | Nerve Head | |  |  |  |  |  |  |
| (OD = 41) | |  |  |  |  |  |  |  | (OS = 43) | |  |  |  |  |  |  |
|  | Inferior | 0.227 | 0.038 | (0.03 to 0.047) | 16.9% | 0.107 | (0.084 to 0.13) |  |  | Inferior | 0.229 | 0.041 | (0.033 to 0.05) | 18.0% | 0.114 | (0.09 to 0.138) |
|  | Superior | 0.214 | 0.051 | (0.04 to 0.062) | 23.8% | 0.141 | (0.111 to 0.172) |  |  | Superior | 0.196 | 0.035 | (0.028 to 0.042) | 17.9% | 0.097 | (0.076 to 0.117) |
|  | Nasal | 0.208 | 0.053 | (0.042 to 0.065) | 25.7% | 0.148 | (0.116 to 0.18) |  |  | Nasal | 0.218 | 0.047 | (0.037 to 0.057) | 21.7% | 0.131 | (0.103 to 0.159) |
|  | Temporal | 0.159 | 0.061 | (0.048 to 0.074) | 38.5% | 0.169 | (0.133 to 0.206) |  |  | Temporal | 0.151 | 0.066 | (0.052 to 0.08) | 43.7% | 0.183 | (0.144 to 0.222) |
|  | Segment | 0.201 | 0.029 | (0.023 to 0.035) | 14.3% | 0.080 | (0.063 to 0.097) |  |  | Segment | 0.197 | 0.028 | (0.022 to 0.033) | 14.0% | 0.076 | (0.06 to 0.093) |
| RPC | |  |  |  |  |  |  |  | RPC | |  |  |  |  |  |  |
| (OD = 41) | |  |  |  |  |  |  |  | (OS = 43) | |  |  |  |  |  |  |
|  | Inferior | 0.176 | 0.055 | (0.043 to 0.067) | 31.4% | 0.153 | (0.12 to 0.186) |  |  | Inferior | 0.189 | 0.048 | (0.038 to 0.058) | 25.3% | 0.133 | (0.105 to 0.161) |
|  | Superior | 0.129 | 0.049 | (0.038 to 0.06) | 38.1% | 0.136 | (0.106 to 0.165) |  |  | Superior | 0.108 | 0.035 | (0.028 to 0.043) | 32.6% | 0.098 | (0.077 to 0.118) |
|  | Nasal | 0.144 | 0.046 | (0.036 to 0.056) | 32.0% | 0.128 | (0.1 to 0.156) |  |  | Nasal | 0.155 | 0.057 | (0.045 to 0.069) | 36.6% | 0.157 | (0.124 to 0.19) |
|  | Temporal | 0.067 | 0.052 | (0.04 to 0.063) | 77.4% | 0.143 | (0.112 to 0.174) |  |  | Temporal | 0.078 | 0.059 | (0.047 to 0.072) | 75.3% | 0.164 | (0.129 to 0.198) |
|  | Segment | 0.131 | 0.033 | (0.026 to 0.04) | 25.3% | 0.091 | (0.072 to 0.111) |  |  | Segment | 0.133 | 0.028 | (0.022 to 0.034) | 21.0% | 0.078 | (0.061 to 0.094) |
| Disc at choroid level | | |  |  |  |  |  |  | Disc at choroid level | | |  |  |  |  |  |
| (OD = 41) | |  |  |  |  |  |  |  | (OS = 43) | |  |  |  |  |  |  |
|  | Inferior | 0.233 | 0.077 | (0.06 to 0.093) | 33.0% | 0.213 | (0.167 to 0.259) |  |  | Inferior | 0.261 | 0.064 | (0.051 to 0.078) | 24.6% | 0.178 | (0.14 to 0.215) |
|  | Superior | 0.280 | 0.072 | (0.056 to 0.088) | 25.7% | 0.200 | (0.157 to 0.243) |  |  | Superior | 0.291 | 0.054 | (0.042 to 0.065) | 18.4% | 0.149 | (0.117 to 0.18) |
|  | Nasal | 0.235 | 0.078 | (0.061 to 0.095) | 33.2% | 0.217 | (0.17 to 0.264) |  |  | Nasal | 0.254 | 0.059 | (0.046 to 0.071) | 23.1% | 0.163 | (0.129 to 0.197) |
|  | Temporal | 0.236 | 0.070 | (0.055 to 0.085) | 29.5% | 0.193 | (0.151 to 0.235) |  |  | Temporal | 0.231 | 0.057 | (0.045 to 0.069) | 24.6% | 0.158 | (0.125 to 0.191) |
|  | Segment | 0.249 | 0.052 | (0.041 to 0.064) | 21.0% | 0.145 | (0.114 to 0.177) |  |  | Segment | 0.262 | 0.035 | (0.028 to 0.043) | 13.5% | 0.098 | (0.077 to 0.119) |

Sw: Within-subject standard deviation; CV: Coefficient of variation is calculated as Sw / overall mean; Repeatability is calculated as 1.96*√2*Sw; C.I: Confidence Interval

Overall mean: The mean of the scan 1 & 2
